# Factors Affecting Human Papillomavirus Vaccination in Men: Systematic Review

**DOI:** 10.2196/34070

**Published:** 2022-04-26

**Authors:** Hyunjeong Shin, Songi Jeon, Inhae Cho, HyunJi Park

**Affiliations:** 1 College of Nursing Korea University Seoul Republic of Korea; 2 Department of Nursing Catholic Kwandong University Gangneung Republic of Korea; 3 C&R Research Seoul Republic of Korea

**Keywords:** health service use, men, papillomavirus, papillomavirus vaccines, systematic review, vaccination, vaccine, HPV, review, gender

## Abstract

**Background:**

Despite the high risks associated with human papillomavirus (HPV), the HPV vaccination rate of men is far lower than women. Most previous review studies have focused on female vaccination and related affecting factors. However, previous studies have reported that the factors affecting HPV vaccination differ by gender.

**Objective:**

The aim of this review was to identify the factors affecting HPV vaccine initiation in men through a systematic review approach.

**Methods:**

A literature review was conducted across 3 central electronic databases for relevant articles. A total of 30 articles published between 2013 and 2019 met the inclusion criteria and were reviewed in this study.

**Results:**

In total, 50 factors affecting HPV vaccination in men were identified, including 13 sociodemographic factors and social structure factors, 12 belief-related variables, 4 family factors, 4 community factors, 14 variables related to needs, and 3 environmental factors.

**Conclusions:**

To increase HPV vaccination rates in men, strategies targeting young males and their families should consider frequent visits to or contact with health care providers so that health care professionals can provide recommendations for HPV vaccination.

## Introduction

### Background

Human papillomavirus (HPV) infection is one of the most common sexually transmitted infections (STIs) in all genders [1,2]. The available data show that approximately 13 million people are diagnosed with HPV infections annually in the United States [3], and these infections range from genital warts to cancers of the cervix, penis, anus, and head and neck. Some studies reported that HPV infection is associated with 5.2% of newly diagnosed cancers worldwide [4]. However, despite the high incidence of and risks associated with HPV-related diseases for both sexes [3-5], globally, the control of HPV infection is commonly considered for women only.

A crucial strategy for protecting people against HPV is the prophylactic HPV vaccination, which can prevent initial HPV infection [6-8]. Population uptake of HPV vaccination has demonstrated significant effectiveness in preventing HPV-related diseases, such as cervical intraepithelial neoplasia [9]. The World Health Organization has recommended HPV vaccination as a routine immunization for girls and female adolescents [6], and most countries have implemented national HPV vaccination programs for women [10]. More recently, HPV vaccination programs inclusive of all genders have been discussed as being more effective for successfully acquiring herd immunity against HPV [10-12]. However, few countries have included young males in their national HPV vaccination programs [2,10,13]. According to a review that reported the HPV vaccination rates in adolescents, only the United States and Canada have reported rates in male adolescents, and these HPV vaccination rates (1.1%-31.7%) in males are confirmed to be very low compared to females (2.4%-94.4%) [14].

To increase the uptake of HPV vaccination among males, it is essential to understand the factors affecting HPV vaccine initiation. Previous review studies have reported that race and ethnicity, age, health insurance status, previous vaccination history, personal knowledge and awareness of HPV, and parental knowledge and education levels are significant predictors for HPV vaccination uptake [15,16]. Nevertheless, most of the published research to date has focused on female vaccination and the factors affecting it. Although some studies have reported that the factors affecting HPV vaccination differ by gender [17,18], they have rarely identified specific evidence regarding the factors affecting male HPV vaccination. In addition, those results that have reported factors affecting male HPV vaccination have been inconsistent. For example, regarding age, Thomas and colleagues reported that younger males had a higher rate of vaccination [19]. Conversely, other research has shown that it was older participants who had a higher rate of vaccination [20].

Increasing HPV vaccination rates in men requires identifying the unique factors affecting men's HPV vaccine initiation and establishing public health policies accounting for those factors. The Behavioral Model of Health Service Use (BMHSU) offers a multidimensional explanation about a person's use of health services. This study uses the BMHSU to structure the factors influencing HPV vaccination in men and provide a comprehensive understanding of these factors. Therefore, this study aimed to (1) confirm the factors affecting HPV vaccination in men through a systematic review of previous studies and (2) identify which components of the BMHSU were explored and influenced HPV vaccination in men.

### BMHSU Framework

This study uses the BMHSU developed by Andersen [21] as a framework to explain the factors associated with HPV vaccination in men. The BMHSU has been widely used in studies on a person's access to health care and is a reliable model that could help understand determinants of health service usage. Since Andersen's behavioral model was introduced, it has been used to reveal barriers or determinants to accessing medical care in various medical situations, from mental health to cancer screening [22-25]. Hence, it is possible to explain differences in access to health care using model components. Moreover, this model provides a comprehensive and multifaceted understanding of access to health services in several review studies [26,27].

The BMHSU explains that health behaviors can be influenced by individual characteristics and the surrounding environment. In this model, the domain of population characteristics includes predisposing characteristics, enabling resources, and needs. Predisposing characteristics are described as personal propensities reflecting sociodemographic factors (eg, age, gender), social structure (eg, education, race, family size), and beliefs (eg, attitudes, knowledge, values). The enabling resources refer to the means that individuals have available to them, such as insurance, income, and community facilities. The needs refer to personally perceived and evaluated needs. Additionally, the domain of environment includes the health care system and external environment.

## Methods

This study is a systematic review conducted to identify the factors influencing HPV vaccination in men. This study was performed according to the PRISMA (Preferred Reporting Items for Systematic Reviews and Meta-Analyses) guidelines [28]; however, this protocol was not registered.

### Search Strategy

The data search was performed comprehensively across three electronic databases: PubMed, Embase, and CINAHL complete. Articles from the inception of the databases until July 2020 were searched. We applied search terms according to our research question: “What factors affect men's HPV vaccination uptake?” In accordance with the search terms, the MeSH (Medical Subject Headings), Emtree, and free terms were collected from the relevant literature and electronic databases before the search. A keyword search strategy was developed with the following terms: “Papillomavirus Infections,” “Papillomavirus,” “Human Papillomavirus,” “Wart virus,” “HPV,” “Vaccination,” “Vaccine,” “Immunization,” “Male,” “Men,” and “Boy.” The search strategies are given in detail in Multimedia Appendix 1.

### Eligibility Criteria

We set the inclusion criteria for identifying publications in accordance with the purpose of the review, as follows: (1) studies involving heterosexual male participants, (2) availability of HPV vaccine initiation data of the participants, (3) studies reporting factors or predictors associated with the HPV vaccination rate of men, and (4) peer-reviewed articles. The exclusion criteria were as follows: (1) studies presenting data excluding gender classification, (2) gray literature such as conference abstracts, (3) articles not published in English, and (4) articles describing experimental studies. All the articles were cross-checked by the research team.

### Data Extraction and Synthesis

**Figure 1:**
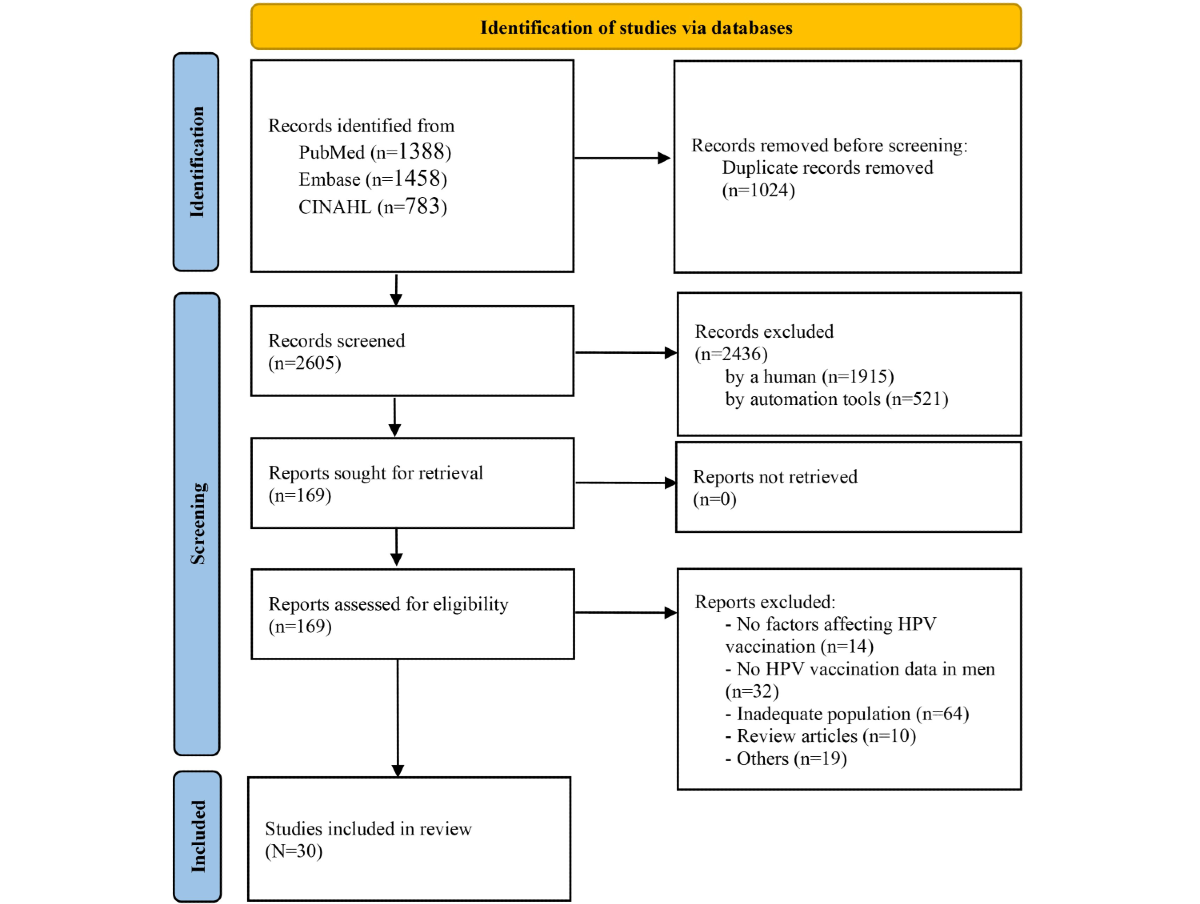
Article selection flow diagram for the systematic review. HPV: Human papillomavirus.

The data were extracted independently by 4 authors (HS, SJ, IC, and HJP). Duplicates were removed first using bibliography software (Endnote Version X9.1, Clarivate Analytics). After removing duplicates, the titles and abstracts were screened using the preset criteria, and irrelevant articles were excluded. A total of 30 studies were finally selected after assessing full-text articles. Figure 1 shows the flow diagram of article selection. In case of a discrepancy over selection, we first tried to reach an agreement through discussion. If the discrepancies remained unresolved, the principal investigator (HS) made the final decision.

For synthesis of the extracted data, the included studies were respectively coded using a predesigned template comprising the study authors, year of publication, country of data collection, study type, data source, age of the participants, sample size, and study results. The factors affecting HPV vaccination in men were classified as the key results. Factors were recorded for every association between factors and any corresponding *P* values used to identify which influencing factors had been studied and where the evidence was statistically significant were recorded. This information was then integrated to review the effects of different factors across the literature. Then, all the factors identified were grouped reflecting the components of the BMHSU.

### Study Quality Assessment

We used the Mixed Methods Appraisal Tool (MMAT) version 2018 for quality assessment of the eligible articles [29]. The MMAT was designed to assess the quality of a variety of studies including qualitative, quantitative, and mixed-method research designs. Each quality criterion consisted of the following five items: (1) whether the sampling strategy was relevant to address the research question; (2) whether the sample was representative of the target population; (3) whether the measurements were appropriate; (4) whether there were risks of bias or confounders; (5) whether the statistical analysis was appropriate to answer the research question. Each item had “Yes,” “No,” or “Cannot tell” as the responses. To evaluate the quality of the studies, we determined the number of “Yes” responses to the items. The quality of the articles was assessed independently by 3 authors (HS, SJ, and HJP). The authors continuously discussed differences in evaluation until an agreement was reached.

## Results

### Study Characteristics

A total of 30 studies were included in the review [17,18,30-57]. The characteristics of the included studies are presented in Table 1. The studies were published between 2013 and 2019. All of them were conducted in the United States except for 1 study that was conducted in Denmark. Of the 30 studies, 26 were cross-sectional studies; the rest were cohort studies (n=3) and longitudinal studies (n=1). The participants of the studies were boys and male adults aged 9 to 34 years. The sample size of each study ranged from 168 to 809,656. The study quality scores obtained using the MMAT ranged from 2 to 5; the quality scores of most studies (n=24) were evaluated as very high (score=5) or high (score=4), 5 studies were rated moderate (score=3), and only 1 was rated low (score=2). The quality scores and the funding sources of the reviewed studies are presented in Table 1 and Multimedia Appendix 2.

**Table 1:** Characteristics of the reviewed studies (N=30)^a^.

Study	Study type	Data source	Age (years)	Sample size (N)	Quality appraisal score^b^
Adjei Boakye et al (2018) [30]	Cross-sectional	2014-2015 National Health Interview Survey	18-26	7588	5
Adjei Boakye et al (2019) [31]	Cross-sectional	2014-2017 National Health Interview Survey	18-34	14,056	5
Agawu et al (2015) [32]	Cohort	2009-2013 large primary care network	11-18	58,757	5
Agénor et al (2015) [33]	Cross-sectional	2013-2014 National Health Interview Survey	18-31	6812	5
Bernat et al (2013) [34]	Cross-sectional	2010 web-based survey	18-24	1682	3
Bollerup et al (2017) [35]	Cohort	2006-2014 Civil Registration System	9-26	809,656	5
Burdette et al (2017) [36]	Cross-sectional	2008-2013 National Immunization Survey-Teen	13-17	56,632	5
Charlton et al (2017) [37]	Cohort	1996-2014 Growing Up Today Study	14-27	3342	3
Choi et al (2016) [17]	Cross-sectional	2012-2013 National Immunization Survey-Teen	13-17	20,355	5
Clarke et al (2016) [38]	Cross-sectional	2012-2013 Electronic medical records from Johns Hopkins Community Physicians clinics	11-26	14,688	5
Daniel-Ulloa et al (2016) [39]	Cross-sectional	2013 National Health Interview Survey	18-30	3003	5
Dela Cruz et al (2018) [40]	Cross-sectional	2014 population-based telephone survey in Hawaii	11-18	Parents (n=799) and their sons (n=467)	3
Fuller and Hinyard (2017) [41]	Cross-sectional	2013 Behavioral Risk Factor Surveillance System	18-26	1624	5
Hechter et al (2013) [42]	Cross-sectional	2009-2010 electronic health record from Kaiser Permanente Southern California	9-17	254,489	5
Johnson et al (2017) [18]	Cross-sectional	2013 National Immunization Survey-Teen	13-17	9554	5
Kepka et al (2016) [43]	Cross-sectional	2012 National Immunization Survey-Teen	13-17	10,141	5
Landis et al (2018) [44]	Cross-sectional	2014 National Immunization Survey-Teen	13-17	10,743	5
Lu et al (2013) [45]	Cross-sectional	2010 National Health Interview Survey	18-26	1741	5
Lu et al (2019) [46]	Cross-sectional	2011-2016 National Immunization Survey-Teen	13-17	9712	5
Morrow (2019) [47]	Cross-sectional	2013-2017 hospital-based teen health center, health department sexually transmitted disease clinic, and the general community	13-16	747	3
Pérez et al (2018) [48]	Cross-sectional	2011-2015 National Health Interview Survey	18-32	15,967	5
Ragan et al (2018) [49]	Cross-sectional	2014 self-administered HPV^c^ Vaccine and Decision-Making Behaviors Survey at 2 universities	18-26	168	4
Rahman et al (2015) [50]	Cross-sectional	2011 National Immunization Survey-Teen	13-17	12,328	5
Ratanasiripong (2015) [51]	Cross-sectional	2012 web-based survey in California	18-26	189	2
Reiter et al (2013) [52]	Longitudinal	2010-2011 internet-based survey from a national sample of parents with sons	11-17	Parents (n=327) and their sons (n=228)	3
Reiter et al (2014) [53]	Cross-sectional	2010-2012 National Immunization Survey-Teen	13-17	4238	5
Sanders Thompson et al (2017) [54]	Cross-sectional	2012 National College Health Assessment IIb Survey	18-26	5013	5
Thompson et al (2016) [55]	Cross-sectional	2009-2013 National College Health Assessment II	18-26	31,130 (28.9% of 107,716)	5
Thompson et al (2019) [56]	Cross-sectional	2016 National Health Interview Survey	18-26	1714	4
Vu et al (2019) [57]	Cross-sectional	2016 data from Project DECOY	18-25	845	4

^a^Except for the study by Bollerup et al [35] that was carried out in Denmark, all the others were conducted in the United States.

^b^ Scores were determined using the MMAT. All scores are quantitative.

^c^HPV: human papillomavirus.

### Factors Affecting HPV Vaccination in Men

We adopted Anderson's BMHSU to structure the results of the review and reveal the relationships among the factors identified. We grouped the identified 50 factors into two components of the BMHSU: 47 population characteristics and 3 environmental factors. The factors identified in this review are presented in Textbox 1 and Table 2.

**Summary of the factors identified in this review. HPV: human papillomavirus; STI: sexually transmitted infection; Pap smear: Papanicolaou smear.**

**Population characteristics (47 factors)**

- **Predisposing (25 factors)**
  - **Sociodemographic and social structure factors (13 factors)**
    - Race and ethnicity
    - Age
    - Parental education level
    - Sexual orientation
    - Nativity status
    - Relationship and marital status
    - Parental age
    - Parental marital status
    - Education level
    - Employment status
    - Number of children in household
    - Language proficiency of caregivers
    - Parental employment status
  - **Beliefs (12 factors)**
    - Parental awareness of HPV vaccine
    - Attitude toward HPV vaccination
    - Awareness of HPV or HPV vaccine
    - Perceived behavioral control
    - Receipt of STI information
    - Searching for health information
    - Subjective norms about getting HPV vaccine
    - Parents’ perceived effectiveness of HPV vaccine
    - Parents’ perceived risks of HPV
    - Parental talks with sons about HPV vaccine
    - Parental willingness to get sons free HPV vaccine
    - Parents’ perceived severity of HPV-related cancers
- **Enabling (8 factors)**
  - **Family (4 factors)**
    - Household income
    - Insurance type
    - Having clinics for usual health care
    - Current state of insurance
  - **Community (4 factors)**
    - Region
    - Size and type of educational institution
    - Medical accessibility
    - Availability of cost-free vaccination programs
- **Need (14 factors)**
  - **Perceived (13 factors)**
    - Vaccinations other than HPV
    - Number of visits to clinics
    - Sexual behaviors
    - Time from the last check-up visit
    - Perceived personal health status
    - HIV testing history
    - Check-up at 11-12 years (eg, well-child visit)
    - History of urinary tract infections
    - Cigarette smoking
    - Reasons for clinic visits (preventive or acute)
    - Maternal vaccination for influenza
    - Maternal STI history
    - Maternal Pap smear screening
  - **Evaluated (1 factor)**
    - Mother’s abnormal Pap smear result

**Environment (3 factors)**

- **Health care system (3 factors)**
  - Health care provider’s recommendation for HPV vaccine
  - Year that vaccinations were performed
  - Facility type

**Table 2:** Studies researching factors affecting human papillomavirus vaccination in men.

Factors					References		Number of studies
**Population characteristics**							
	**Predisposing**						
		**Sociodemographic and social structure factors**					
			Race and ethnicity	[17^a^,^18^^a^,^30^,^34^^a^,^36^^a^,^38^^a^,^39^,^40^^a^,^41^^a^,^43^^a^,^44^^a^,^45^,^46^^a^,^48^,^49^,^52^^a^,^53^,^54^^a^,^55^,^57^^a^]		20	
			Age	[17^a^,^18^,^30^^a^,^34^,^36^^a^,^38^^a^,^40^^a^,^43^,^46^^a^,^47^^a^,^48^,^49^,^51^^a^,^54^,^55^^a^,^57^]		16	
			Parental education level	[17^a^,^18^,^35^^a^,^36^^a^,^40^,^43^^a^,^44^^a^,^46^^a^,^53^,^57^]		10	
			Sexual orientation	[33^a^,^34^,^37^^a^,^39^,^54^^a^,^55^,^57^]		7	
			Nativity status	[30,31^a^,^35^^a^,^46^,^48^^a^,^53^,^57^]		7	
			Relationship and marital status	[18,36^a^,^40^,^43^,^44^,^46^^a^]		6	
			Parental age	[30,34,45,48^a^,^54^,^55^^a^]		6	
			Parental marital status	[18^a^,^35^^a^,^36^^a^,^43^,^44^^a^,^46^]		6	
			Education level	[18,30^a^,^41^,^45^,^48^^a^]		5	
			Employment status	[30,45,48^a^,^54^^a^,^56^]		5	
			Number of children in household	[36,53]		2	
			Language proficiency of caregivers	[18^a^,^53^]		2	
			Parental employment status	[35]^a^		1	
		**Beliefs**					
			Parental awareness of HPV^b^ vaccine	[40,50]^a^		2	
			Attitude toward HPV vaccination	[51]^a^		1	
			Awareness of HPV or HPV vaccine	[45]^a^		1	
			Perceived behavioral control	[51]^a^		1	
			Receipt of STI^c^ information	[54]^a^		1	
			Searching for health information	[56]^a^		1	
			Subjective norms about getting HPV vaccine	[51]		1	
			Parents’ perceived effectiveness of HPV vaccine	[40]^a^		1	
			Parents’ perceived risks of HPV	[40]^a^		1	
			Parental talks with sons about HPV vaccine	[52]		1	
			Parental willingness to get sons free HPV vaccine	[52]		1	
			Parents’ perceived severity of HPV-related cancers	[40]		1	
	**Enabling**						
		**Family**					
			Household income	[18,35^a^,^36^^a^,^41^,^43^,^44^^a^,^45^^a^,^46^,^49^,^53^^a^,^56^]		11	
			Insurance type	[17^a^,^38^^a^,^43^^a^,^44^^a^,^45^,^46^^a^,^47^^a^,^53^,^54^^a^]		9	
			Having clinics for usual health care	[30,31,41^a^,^45^,^48^,^56^]		6	
			Current state of insurance	[18,30,31,41,48^a^,^54^^a^]		6	
		**Community**					
			Region	[18,30,36,41,44,48,54]^a^		7	
			Size and type of educational institution	[49,54^a^,^57^]		3	
			Medical accessibility	[38,46]^a^		2	
			Availability of cost-free vaccination programs	[18]^a^		1	
	**Need**						
		**Perceived**					
			Vaccinations other than HPV	[32^a^,^43^^a^,^44^^a^,^49^,^54^^a^]		5	
			Number of visits to clinics	[30,31,32,38,46]^a^		5	
			Sexual behaviors	[34^a^,^37^^a^,^45^,^49^]		4	
			Time from the last check-up visit	[18^a^,^53^^a^,^56^]		3	
			Perceived personal health status	[32^a^,^54^]		2	
			HIV testing history	[34,41]^a^		2	
			Check-up at 11-12 years (eg, well-child visit)	[44,46^a^]		2	
			History of urinary tract infections	[54]^a^		1	
			Cigarette smoking	[47]^a^		1	
			Reasons for clinic visits (preventive or acute)	[32]^a^		1	
			Maternal vaccination for influenza	[42]^a^		1	
			Maternal STI history	[42]		1	
			Maternal Pap smear^d^ screening	[42]^a^		1	
		**Evaluated**					
			Mother’s abnormal Pap smear result	[42]		1	
**Environment**							
	**Health care system**						
		Health care provider’s recommendation for HPV vaccine			[17,18,36,44,46,50,53,57]^a^		8
		Year when vaccinations were performed			[17,32,36,53]^a^		4
		Facility type			[18,43,46]		3

^a^Studies that obtained statistically significant findings (*P*<.05).

^b^HPV: human papillomavirus.

^c^STI: sexually transmitted infection.

^d^Pap smear: Papanicolaou smear

#### Population Characteristics

According to the BMHSU, the domain of population characteristics includes predisposing factors, enabling factors, and need factors.

##### Predisposing Factors

Among the predisposing factors, social structure reflected the social position of the individuals in their society. We combined sociodemographic factors and social structure components of the BMHSU because it may be difficult to distinguish them in different cultural contexts. We then found 13 factors that were categorized as sociodemographic factors and social structure factors, including age, sexual orientation, relationship or marital status, parental age, race or ethnicity, education level, employment status, nativity status, number of children in the household, parental education level, parental marital status, language proficiency of caregivers, and parental employment status. Approximately half of the studies (n=16) explored age as a factor related to the HPV vaccination initiation [17,18,30,34,36,38,40,43,46-49,51,54,55,57]. Among them, 9 studies showed significant associations between age and vaccination [17,30,36,38,40,46,47,51,55]. However, the 5 studies with teenagers reported more vaccinations at older ages [17,36,40,46,47], whereas the other 4 studies covering populations aged up to 26 years reported the opposite relationship [30,38,51,55]. The most frequently mentioned factor was race or ethnicity (n=20) [17,18,30,34,36,38-41,43-46,48,49,52-55,57]; of the 20 studies, 13 showed significant relationships between race or ethnicity and vaccination [17,18,34,36,38,40,41,43,44,46,52,54,57]. However, some of the results are inconsistent, with 7 studies reporting that Hispanics were more vaccinated than non-Hispanics [17,18,36,43,44,46,54]; other studies reported conflicting results that African Americans [34,38] were more vaccinated or Caucasians [40,52] were less vaccinated than other ethnic groups. As for parental education level, 6 out of 10 studies revealed significant associations [17,35,36,43,44,46]. The association between parents’ marital status and HPV vaccination was explored in 6 studies [18,35,36,43,44,46]. A significant association was reported in 4 of them [18,35,36,44], and married parents were less likely to have their sons vaccinated according to 3 studies [18,36,44], but 1 study in Denmark reported the opposite result [35].

We identified 12 belief factors, namely attitude toward HPV vaccination, awareness of HPV or HPV vaccine, perceived behavioral control, receipt of STI information, searching for health information, subjective norms about getting HPV vaccine, parental awareness of HPV vaccine, parents’ perceived effectiveness of HPV vaccine, parents’ perceived risks of HPV, parental talks with sons about HPV vaccine, parental willingness to get sons vaccinated, and parents’ perceived severity of HPV-related cancers. Beliefs refer to individual values about health, attitudes toward health services, or knowledge about disease. Significantly, sons of parents with high awareness of the HPV vaccines were more likely to be vaccinated [40,50]. However, other significant factors belonging to the belief domain were reported in only 1 study.

##### Enabling Factors

We found four enabling factors that were ultimately categorized as family factors: household income, health insurance types, having clinics for usual health care, and current state of insurance. As for household income, those with low economic status had a higher likelihood of being vaccinated according to 4 studies [36,44,45,53]; however, 1 study conducted in Denmark reported conflicting results [35]. Regarding the relationships between insurance type and HPV vaccination, 7 out of 9 studies reported that men with Medicaid or public insurance were significantly more likely to get vaccinated than men with private insurance [17,38,43,44,46,47,54]. In addition, uninsured people were less likely to take HPV vaccinations [48,54].

The remaining four factors identified were categorized as community factors: region (eg, specific city or state), size and type of educational institution, accessibility to medical facilities, and availability of cost-free vaccination programs. The rate of HPV vaccination was significantly higher in urban areas [38,46] where accessibility to medical facilities was better.

##### Need Factors

The need factors of populations could be classified into perceived and evaluated. In this study, 13 factors were identified as perceived needs that affect HPV vaccination in men, including vaccinations other than HPV, the number of visits to clinics, sexual behaviors, time from the last check-up visit to a clinic, perceived personal health status, history of urinary tract infections, participants’ HIV testing history, check-up at 11 to 12 years (eg, well-child visit), cigarette smoking, reasons for clinic visits, maternal vaccination for influenza, maternal STI history, and maternal Papanicolaou (Pap) smear screening. The reviewed studies reported that men who received other vaccinations (eg, influenza; meningitis; hepatitis B; and tetanus, diphtheria, and pertussis vaccines) were more likely to take HPV vaccines [32,43,44,54]. All 5 studies that explored the number of visits to clinics as the factors affecting HPV vaccination in men [30-32,38,46] consistently reported that more frequent clinic visits had a significant relationship with more HPV vaccinations. It was also found that more HPV vaccinations were given to those who had undergone HIV tests [34,41] and those whose mothers had undergone Pap smear tests [42].

In this study, one factor was classified as an evaluated need: the mother’s abnormal Pap smear result. It was found that initiation of HPV vaccination was associated with abnormal maternal Pap smear results [42].

#### Environment

The BMHSU suggests the health care system and external environment as the environmental factors that affect individual health behaviors [21]. This review did not reveal any significant external environmental factors. The following were identified as health care system factors: health care provider’s recommendations for HPV vaccination, the year that the vaccinations were performed, and the types of health care facilities that men usually use. The most frequently reported factor was the health care provider’s recommendations for HPV vaccination (n=8). Those who received recommendations for HPV vaccination showed more HPV vaccination uptake [17,18,36,44,46,50,53,57]. The studies also revealed that HPV vaccination rates have gradually increased since the year when the government recommended HPV vaccination for males in the United States [17,32,36,53].

## Discussion

### Principal Findings

This systematic review identified 30 peer-reviewed research articles published from 2013 to 2019 that contained studies analyzing the factors affecting male HPV vaccination. We identified a total of 50 modifiable and nonmodifiable factors across a wide range of domains assessed by the authors of those studies as being associated with male HPV vaccination. For this study, we used Andersen’s BMHSU to structure the identified factors. The highest rates of vaccination tended to be associated with the following identified factors: men aged 10 to 20 years, Hispanic race, adolescents with single parents, higher parental knowledge and awareness of HPV vaccination, low economic status, individuals with public insurance, males living in urban areas, individuals receiving other vaccinations, frequent visits to clinics, and receiving health care providers’ recommendations. Although many factors have been identified in previous studies and could be potential affecting factors (eg, attitude toward HPV vaccination, parental perceptions of the risks of HPV), they were often only assessed by single studies featuring small sample sizes, thus limiting the generalizability of these factors to the larger male population.

We found that health care providers’ recommendations are major facilitators of HPV vaccinations in men. These findings coincided with those of prior review studies noting that the health care provider’s recommendation was the most significant factor leading to the initiation of HPV vaccination [58-60]. One of the studies reviewed in this work reported that receiving the health care provider’s recommendation was the most influential factor in parental decisions on vaccinating children for HPV [59]. Another review study reported that people who were advised to get HPV vaccinations by health care providers were more likely to be vaccinated than people with no recommendations, with an odds ratio of 10.1 [60]. However, despite positive evidence of health care providers' recommendations, the delivery of recommendations associated with HPV vaccine in men is a challenge that must be overcome. Some studies explain a gap associated with recommendations according to sex that originated from the provider's lack of knowledge or opinions [61,62]. Perkins and colleagues also reported that a provider-focused intervention targeting health care providers improved the initiation of the HPV vaccine prominently for boys [63]. Thus, a first step to enhance the rates of male HPV vaccination could be to induce consistent recommendations for immunization from the health care providers through training for health professionals.

This review suggests that frequent contact with health care providers is a promising factor for promoting HPV vaccination rates in men. We found that the number of visits to clinics and accessibility to health care facilities have significant relationships with the initiation of HPV vaccinations in men [30-32,38,46,54]. Furthermore, studies revealed that those males who had taken vaccinations other than those for HPV (eg, tetanus, diphtheria, and pertussis; meningitis; influenza; and hepatitis B) had higher HPV vaccination rates. This might have shown the potential of a program developed to link the initiation of HPV vaccination to other vaccinations conducted at the same age [13]. Thus, we should seek frequent contact with health care professionals and explore feasible programs at the same time for men based on these results.

This review also suggests that parental awareness of HPV vaccination is an essential facilitator of HPV vaccinations in children. Several previous studies on men’s HPV vaccinations have noted that a lack of knowledge regarding the HPV vaccine is one of the causes of low HPV vaccination rates in men [64-66]. Radisic and colleagues also reported that parental awareness of HPV was strongly associated with vaccinating children [67]. Because it is advisable to initiate vaccination for early adolescents aged 9 to 14 years [13], it is important to increase parental awareness of vaccination after the national introduction of HPV vaccination in men. Thus, these findings suggest the necessity of parental education to promote HPV vaccination in men effectively. To promote rates of HPV vaccine uptake in men, national policies should include parents as decision-makers and aim to increase their knowledge and awareness.

In this study, some factors (eg, household income, marital status of parents) were significant but inconsistent in their direction of association with male HPV vaccination. In a study performed in the United States, household income had a negative relationship with HPV vaccination, whereas in a study performed in Denmark, a positive one was found. Regarding ethnic groups, the reviewed studies suggest various ethnic groups to be the most vaccinated, for example, Hispanics or African Americans. One of the reasons is that most of the reviewed studies were conducted in the United States. Since 2011, the United States has included men in the national HPV vaccination program. Accordingly, the HPV vaccination rate in men has changed over time, and it can be expected that the findings are diverse according to the time of the studies included in this review. For more nuanced and conclusive results on these aspects, future research should be performed in a wider range of countries and target families with different socioeconomic statuses and ethnic backgrounds.

### Limitations

This review has some limitations. First, most of the included studies, except for 1, were conducted in the United States. Despite the comprehensive findings on the factors affecting male HPV vaccination, it might be difficult to generalize these results. Second, most studies that were reviewed in this work performed secondary data analyses and extracted the variables from available large-scale data sets that had already been constructed for different purposes. Thus, a wide range of psychosocial variables or factors were not reflected in the reviewed studies. Third, we only included heterosexual participants. Men who have sex with men (MSM) are at high risk for HPV infection [68,69]. HPV vaccination was emphasized for the MSM population more than for heterosexual males, as seen in the differences with respect to the recommended age [70]. For this reason, we assumed that the factors affecting HPV vaccine initiation in men might be different in the case of MSM. However, further research should include various sexual orientations and gender groups to understand and unify disparities in HPV vaccine initiation in men.

### Conclusions

This review analyzed 30 research articles using a systematic approach and identified a total of 50 factors affecting HPV vaccination in men. Based on our results, to increase the rates of male HPV vaccination, strategies targeting health care providers should be considered so that health care professionals can provide consistent and evidence-based recommendations for HPV vaccination, including frequent visits or contact with health care providers. In addition, national policies should include parents as decision-makers and increase their knowledge and awareness of HPV vaccination and its effects on preventing a variety of cancers.

## Appendix

Multimedia Appendix 1 Search strategy used in electronic databases.

Multimedia Appendix 2 Funding sources of the reviewed studies (N=30).

## Abbreviations

BMHSU: Behavioral Model of Health Services Use
HPV: human papillomavirus
MMAT: Mixed Methods Appraisal Tool
MSM: men who have sex with men
Pap smear: Papanicolaou smear
STI: sexually transmitted infection
