# Supplementary material for: Factors Affecting Human Papillomavirus Vaccination in Men: Systematic Review
Source: JMIR Public Health Surveill. 2022 Apr 26;8(4):e34070. doi: 10.2196/34070 (PMC9092232; doi:10.2196/34070)
Supplement: Multimedia Appendix 1 [file publichealth_v8i4e34070_app1.docx]

**Multimedia Appendix 1. Electronic Search strategy in electronic databases**

| **PubMed**  (((("papillomavirus infections"[Title/Abstract] OR "Papillomavirus"[Title/Abstract]) OR "human papillomavirus"[Title/Abstract]) OR "HPV"[Title/Abstract]) AND ("Vaccination"[Title/Abstract] OR "vaccine*"[Title/Abstract])) AND ("Male"[Title/Abstract] OR "Men"[Title/Abstract])  Results : 1,388 (20.07.14.)  **Embase**  ('wart virus'/exp OR 'wart virus'/de OR (('wart'/exp OR 'wart'/de) AND ('virus'/exp OR 'virus'/de)) OR 'wart virus infection'/exp OR 'wart virus infection'/de OR (('wart'/exp OR 'wart'/de) AND ('virus'/exp OR 'virus'/de) AND ('infection'/exp OR 'infection'/de)) OR 'hpv'/exp OR 'hpv'/de) AND (vaccine* OR 'vaccination'/exp OR 'vaccination'/de OR 'immunization'/exp OR 'immunization'/de) AND ('male'/exp OR 'male'/de OR 'boy'/exp OR 'boy'/de)  Results : 1,458 (20.07.14.)  **CINAHL complete**  (TI ( (Papillomavirus Infections OR Papillomavirus OR Human Papillomavirus OR HPV) AND (Vaccination OR Immunization OR Vaccine*) AND (Male OR Men) )) OR (AB ( (Papillomavirus Infections OR Papillomavirus OR Human Papillomavirus OR HPV) AND (Vaccination OR Immunization OR Vaccine*) AND (Male OR Men) ))  Results : 783 (20. 07. 14.) |
| --- |
