# Supplementary material for: Factors Affecting Human Papillomavirus Vaccination in Men: Systematic Review
Source: JMIR Public Health Surveill. 2022 Apr 26;8(4):e34070. doi: 10.2196/34070 (PMC9092232; doi:10.2196/34070)
Supplement: Multimedia Appendix 2 [file publichealth_v8i4e34070_app2.docx]

**Multimedia Appendix 2. The funding source of the reviewed studies (N=30)**

| **Study** | **Funding source** |
| --- | --- |
| Adjei Boakye et al (2018) [30] | No financial support |
| Adjei Boakye et al (2019) [31] | No financial support |
| Agawu et al (2015) [32] | Center of Excellence for Diversity in Health Education and Research at the University of Pennsylvania, and Health Resources and Services Administration |
| Agénor et al (2015) [33] | National Institutes of Health, Society of Family Planning, Lesbian Health Fund, National Cancer Institute, and Maternal and Child Health Bureau. |
| Bernat et al (2013) [34] | National Institutes on Drug Abuse and National Institutes of Mental Health |
| Bollerup et al (2017) [35] | No financial support |
| Burdette et al (2017) [36] | No financial support |
| Charlton et al (2017) [37] | Eunice Kennedy Shriver National Institute of Child Health and Human Development, National Institutes of Health, Health Professionals Advancing LGBT Equality's Lesbian Health Fund, and Patient-Centered Outcomes Research Institute |
| Choi et al (2016) [17] | No financial support |
| Clarke et al (2016) [38] | National Institutes of Health and National Cancer Institute |
| Daniel-Ulloa et al (2016) [39] | Centers for Disease Control and Prevention |
| Dela Cruz et al (2018) [40] | National Cancer Institute, National Institute on Minority Health and Health Disparities, and Queen’s Medical Center |
| Fuller and Hinyard (2017) [41] | No financial disclosures |
| Hechter et al (2013) [42] | Department of Research and Evaluation, Kaiser Permanente Southern California |
| Johnson et al (2017) [18] | No financial support |
| Kepka et al (2016) [43] | The University of Utah College of Nursing, the Huntsman Cancer Institute Foundation, the Primary Children's Hospital Foundation, the Beaumont Foundation, and the National Center for Advancing Translational Sciences of the National Institutes of Health |
| Landis et al (2018) [44] | No financial support |
| Lu et al (2013) [45] | No financial disclosures |
| Lu et al (2019) [46] | No financial disclosures |
| Morrow (2019) [47] | No financial disclosures |
| Pérez et al (2018) [48] | National Institutes of Health |
| Ragan et al (2018) [49] | Hubert Department of Global Health at Emory University’s Rollins School of Public Health |
| Rahman et al (2015) [50] | The Office of Research on Women’s Health, the Office of the Director, the National Institute of Allergy and Infectious Diseases, and the National Institutes of Health. |
| Ratanasiripong (2015) [51] | California State University, Dominguez Hills |
| Reiter et al (2013) [52] | Investigator-Initiated Studies Program of Merck Sharp & Dohme Corp, American Cancer Society, National Institutes of Health, Cancer Control Education Program at the University of North Carolina, Chapel Hill, Lineberger Comprehensive Cancer Center, and a National Research Service Award in Primary Medical Care at the University of Minnesota. |
| Reiter et al (2014) [53] | Cervical Cancer-Free America, National Cancer Institute |
| Thompson et al (2017) [54] | No financial disclosures |
| Thompson et al (2016) [55] | No financial disclosures |
| Thompson et al (2019) [56] | No financial disclosures |
| Vu et al (2019) [57] | National Cancer Institute, National Institutes of Health, National Institute for Allergy and Infectious Diseases, and National Institutes of Health |
